# Reconstruction of the Anterior Skull Base Using the Nasoseptal Flap: A Review

**DOI:** 10.3390/cancers16010169

**Published:** 2023-12-29

**Authors:** Michael T. Werner, Desmond Yeoh, Judd H. Fastenberg, Mark B. Chaskes, Aron Z. Pollack, John A. Boockvar, David J. Langer, Randy S. D’Amico, Jason A. Ellis, Brett A. Miles, Charles C. L. Tong

**Affiliations:** 1 Department of Otolaryngology-Head & Neck Surgery, Northwell Health System, New York, NY 11042, USA; mwerner4@northwell.edu (M.T.W.); dyeoh1@pride.hofstra.edu (D.Y.); jfastenberg@northwell.edu (J.H.F.); mchaskes@northwell.edu (M.B.C.); apollack@northwell.edu (A.Z.P.); bmiles4@northwell.edu (B.A.M.); 2 Department of Neurosurgery, Northwell Health System, New York, NY 11042, USA; jboockvar@northwell.edu (J.A.B.); dlanger@northwell.edu (D.J.L.); rdamico8@northwell.edu (R.S.D.); jellis2@northwell.edu (J.A.E.)

**Keywords:** expanded endoscopic endonasal surgery, anterior skull base reconstruction, nasoseptal flap, pedicled flaps, transnasal transsphenoidal pituitary surgery

## Abstract

**Simple Summary:**

Patients with pituitary tumors and other masses at the bottom of the skull are at risk of brain infection and leaks of the fluid surrounding the brain when the tumor is removed surgically. To prevent these types of complications, surgeons have developed techniques to patch the surgical area with normal tissue taken from other parts of the body. In the past, this tissue was taken from the head, neck, and shoulder regions. Newer techniques allow the entire surgery to occur within the nose, resulting in fewer side effects and faster recovery. This includes taking normal tissue from within the nose to repair the base of the skull where the mass is removed. The nasoseptal flap is the most common method for this type of intranasal repair. In this paper, we describe the development and evolution of this surgical technique and discuss the impact that it has had on patient outcomes.

**Abstract:**

The nasoseptal flap is a workhorse reconstructive option for anterior skull base defects during endonasal surgery. This paper highlights the versatility of the nasoseptal flap. After providing a brief historical perspective, this review will focus on the relevant primary literature published in the last ten years. We will touch upon new applications of the flap, how the flap has been modified to expand its reach and robustness, and some of the current limitations. We will conclude by discussing what the future holds for improving upon the design and use of the nasoseptal flap in anterior skull base reconstruction.

## 1. Introduction

Since its initial description in 2006, the nasoseptal flap (NSF) has become a workhorse reconstructive option in the endonasal repair of anterior skull base defects. The flap is pedicled on the posterior septal nasal artery, which branches off the sphenopalatine artery and courses along the posterior nasal cavity and inferior sphenoid face. Due to its versatile nature and customizability, the NSF can be utilized for a range of different defects extending from the frontal recess to the low clivus in the sagittal plane. This paper describes the evolution of the NSF, including its uses, modifications, and relevant morbidity.

## 2. History of Anterior Skull Base and Cerebrospinal Fluid Leak Repairs

Anterior skull base defects can be categorized by anatomic location, size, and etiology. The defect may be spontaneous or surgical (Table 1). Spontaneous defects may derive from purely intracranial pathology, or they may arise from dural tumors or invasive sinonasal malignancies. Elevated intracranial pressure due to idiopathic intracranial hypertension or cerebrospinal fluid (CSF) outflow obstruction may contribute. CSF leaks arising from skull base defects are typically divided into low-flow and high-flow leaks based on whether a ventricle or cistern is involved (high-flow leaks) or when the dural defect is greater than 1 cm (smaller defects are considered low flow) [1,2]. The type of defect and the amount of CSF leak are the primary considerations in planning a skull base reconstruction. 

**Figure 1:**
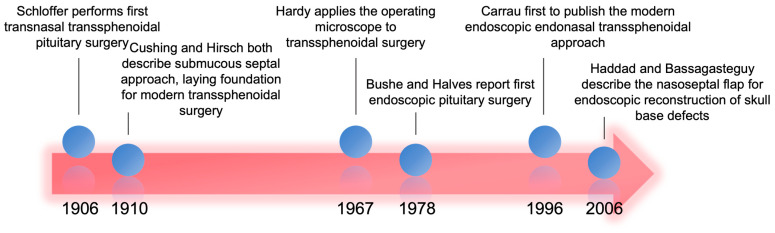
Timeline covering 100 years of milestones leading to modern endoscopic endonasal skull base surgery and use of NSF [17,18].

The field of anterior skull base reconstruction emerged from the transition of pituitary surgery from transcranial approaches toward more minimally invasive transsphenoidal approaches in the 1960s. It further blossomed with the development of the NSF, which was the first vascularized reconstructive option for endonasal endoscopic skull base surgery with a comprehensive description of the blood supply [10,11]. Prior to the transition to transsphenoidal surgery in the early 1900s, pituitary lesions were approached through craniotomies, and patients suffered from historically high mortality rates exceeding 20% [11]. In 1906, the first transsphenoidal technique to remove a pituitary neoplasm was demonstrated (Figure 1). Rapid advances, including the use of sublabial incisions and submucous septal resections, led Harvey Cushing to publish a drastically reduced surgical mortality rate of 5.6% in 1910 [12]. However, the elevated risk of intracranial infection from traversing the mucosal surface, technical improvements in transcranial surgery, and the superior visualization achieved by transcranial surgery all resulted in the neurosurgical world largely abandoning transsphenoidal techniques. 

In the 1960s, a resurgence of a transsphenoidal approach for pituitary neoplasms arose with the improved abilities to visualize the sella, first using the binocular microscope, and then the endoscope. The emergence of the endoscope as the superior tool for visualization during skull base surgery led to the development of new transsphenoidal approaches, including sublabial and endonasal routes. In 1996, Carrau et al. reported the first endoscopic transsphenoidal pituitary surgery [13]. In the early days of endoscopic skull base surgery, the skull base defects encountered by surgeons were predominately located in the sellar or olfactory groove regions. These were relatively small defects with low-flow CSF leaks. Management typically involved grafts composed of fat, fascia, or muscle [14].

To provide some historical context to the surgical management of CSF leaks, the first instance of an intracranial repair of a CSF leak was in 1926, when a bifrontal craniotomy was performed and a fascia lata graft was sutured to repair the dural defect. This technique had a high risk of failure of up to 27% and required brain retraction, which had the possibility of causing injury to sensitive neurovascular structures [15]. To avoid brain retraction and its complications, a naso-orbital extracranial approach to repair leaks was pioneered in 1948 [16]. The success rate was also higher at 86-97% with fewer complications limited to possible orbital injury, transient facial paralysis, and the risk of infection [9]. In 1952, Hirsch performed a transnasal repair of the sphenoid sinus but it was limited in application due to the narrow visual field [17]. With the emergence of the endoscope, that ceased to be the case.

With the expansion of endoscopic endonasal surgeries to techniques to access lesions in the clival, odontoid, suprasellar, and infratemporal regions, surgeons encountered larger, high-flow defects that had historically been seen only during open craniofacial approaches. Initially, repair efforts with free tissue grafts contributed to an incidence of postoperative CSF leaks of approximately 20% [19]. Persistent leaks were generally found in fluid-dependent areas of the graft margin or along the superior border where the graft could migrate [19]. This led to an exploration of various onlay and inlay combinations. Inlay grafts are typically placed deep to the dura with enough surface area to create a wide margin around the defect. These are commonly collagen-based and provide a gasket-like seal due to the outward hydrostatic pressure exerted by CSF. Onlay grafts are layered superficial to the defect on the sinonasal surface of sufficient size to adequately cover a margin around the defect. Onlay grafts are more variable and surgeon-dependent, but can comprise acellular dermis, fat, fascia, or mucosa [14]. These layers bolster coverage over the defect, function as biological dressings to maintain a barrier surface separating the endonasal and intracranial compartments, and promote dural healing and remucosalization. 

Despite the advances in the use of multilayered graft closure, the postoperative CSF leak rate was still estimated to be 20–30% for large defects > 3 cm [10]. As such, many surgeons favored combined endoscopic and open approaches for large CSF leaks so that an external regional vascularized flap, such as the pericranial, galeal, or temporoparietal flaps, could be raised and rotated inward for closure. With the adoption of the NSF, which could be raised endonasally, the postoperative CSF leak improved drastically (discussed below) and allowed surgeons to perform purely endoscopic surgery. To quantify the improvement, a comparison between graft techniques and the use of the NSF demonstrated that the rates of CSF leaks declined from 12.5% to 3% [20]. 

**Figure 2:**
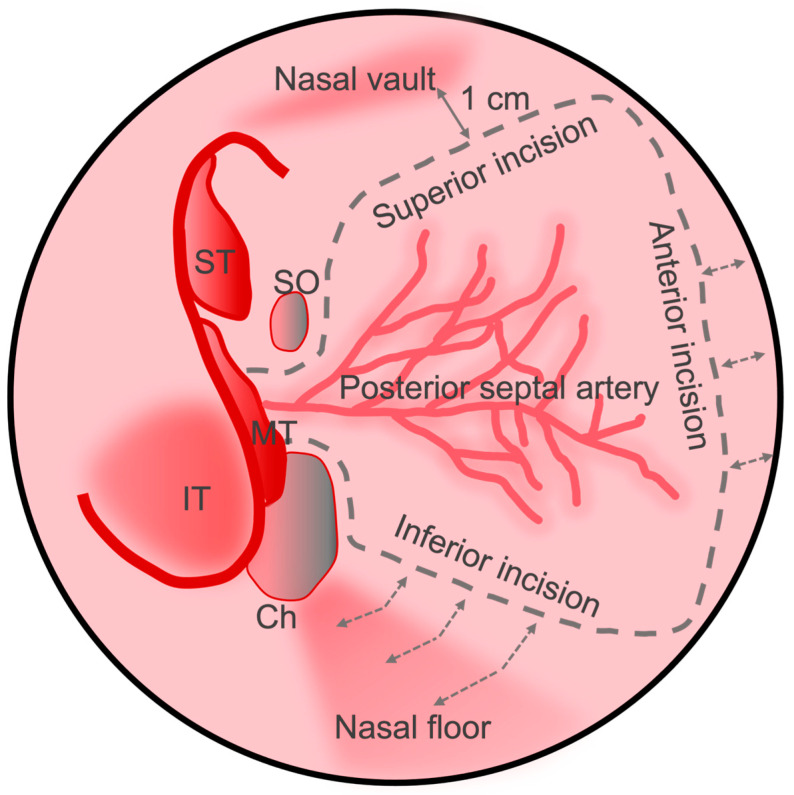
Schematic view of the nasal septum with surgical landmarks, predicted posterior septal artery vascular pedicle distribution, and nasoseptal flap incisions demarcated. The incisions can be carried anteriorly or laterally as depicted by the dashed arrows. IT: inferior turbinate; MT: middle turbinate; ST: superior turbinate; SO: sphenoid ostium; Ch: choana.

In 2006, Hadad et al. first described the NSF in a case series of 43 patients [10]. Originally referred to as the Hadad-Bassagasteguy flap or HBF, the flap is based on the distribution of the posterior septal nasal branch (also referred to as the posterior nasal or posterior septal artery) of the sphenopalatine artery and includes the nasoseptal branch which supplies the majority of the septal mucoperiosteum and mucoperichondrium, and which anastomoses with branches of the anterior ethmoid, greater palatine, and anterior facial arteries. The flap is created by making two parallel incisions along the sagittal plane of the septum (Figure 2). The inferior incision is made along the maxillary crest, while the superior incision is made approximately 1 cm from the superior extent of the septum to protect the olfactory epithelium. A vertical incision is made anteriorly to connect the two. Posteriorly, the superior incision is carried inferolaterally across the sphenoid rostrum parallel with the posterior nasal artery at the level of the natural ostium. The inferior incision is carried across laterally just superior to the choana to create the vascular pedicle. The length of the septal incisions can be adjusted anteriorly according to the size of the defect. Likewise, the inferior incision can be extended laterally along the nasal cavity floor to harvest a wider flap. 

This initial case series describing the flap highlighted its versatility across multiple tumor types and endonasal approaches with only two postoperative CSF leaks reported. Though prior endonasal flaps, such as the middle turbinate and septal flaps, had been used to repair spontaneous CSF leaks and septal perforations, these were based on random blood supply, and their limited arc of rotation often pulled the flap from the defect. Therefore, the NSF, with its major reduction in postoperative CSF leak rate, was a major boon to the expansion in endoscopic approaches for anterior skull base tumors. 

## 3. Impact of Nasoseptal Flap Closure on Postoperative CSF Leaks

Since the original NSF paper, several studies have demonstrated a decline in postoperative CSF leaks when the NSF is used in comparison to traditional grafting techniques. In one study of pituitary skull base surgery, the authors found that the rate of CSF leak in NSF patients was 3% in comparison to 12.5% for those closed with grafts [20]. Likewise, a meta-analysis of post-surgery CSF fluid leaks after skull base meningioma resection reported a decrease from 22% in 2004 to 4% in 2020, which coincides with the widespread adoption of the NSF [21]. When comparing graft reconstruction and the NSF, a review article published in 2012 showed that the leak rate for free grafts was 15.6% in comparison to a 6.7% leak rate for NSFs [22]. In cases of obesity, using a NSF significantly decreased the risk of a CSF leak (from 29.6% to 15.0%), while the difference between using a NSF and a free graft was not significant for patients in a healthy weight category [23]. Another study found that there was an overall success rate of 91.5% when using NSFs but when stratified by location, the differences in leak rates between grafts and NSFs were only significant in the clivus [24].

Several studies have addressed the possible risk factors associated with postoperative CSF leaks after NSF reconstruction (Table 2). In a review of 98 patients, CSF leak incidence was reported as 11%, which was within the expected 5–20% rate reported elsewhere [5]. Abnormal BMI (>25 or <18.5) was the most significant risk factor associated with both CSF leaks and meningitis [23]. Increasing age was also associated with CSF leaks, as was the duration of lumbar drain for meningitis [5]. The mechanism behind a high BMI and increased CSF leak risk is thought to be related to intracranial pressure [25]. Intraoperative CSF leaks and combined endonasal and open craniofacial resections were also risk factors for postoperative leaks [5]. 

Perhaps the most important factor in a postoperative CSF leak after using the NSF is the experience of the surgeon. In a series of 225 consecutive patients in which the NSF was used to repair anterior skull base defects by a single surgeon, the postoperative CSF leak rate was 24% in the first 25 patients and 4% in the last 200 [1]. This highlights the nuance that goes into pre-surgical planning and decision-making about when to implement the nasoseptal flap, as well as intraoperative considerations that mitigate the risk of CSF leaks. Pre-operatively, the most important factor is whether the NSF pedicle may be compromised due to prior surgery or tumor involvement. When the NSF is available, its arc of rotation and size make it a universal reconstructive option regardless of defect size, location, and CSF output. Nevertheless, its success depends on careful dissection without perforating or tearing the mucosa and with particular attention to preserving the vascular bundle and blood supply. This includes careful elevation of the flap inferior to superior and anterior to posterior. Scarring from prior septal surgery, pre-existing perforations, and large septal spurs are the predominant obstacles. In planning the incision, some surgeons opt to map out the arterial supply using a Doppler probe. Postoperatively, most surgeons prefer to pack the nose using a combination of absorbable nasal packing and stents that secure the flap in place. This is preferred over balloons which may exceed the intravascular pressure of the flap, resulting in flap necrosis [11]. NSF necrosis is a relatively rare complication that could result in CSF leaks, epidural empyema, or meningitis. Risk factors include prior surgery which may predispose an individual to poor vascular supply and the use of a fat graft as part of the multilayered closure, which may reflect the need for additional support intraoperatively for a flap found to be less robust. A lack of flap enhancement on a postoperative MRI is an indication that the flap may be necrotic [27]. In cases of flap failure resulting in postoperative CSF leaks, the cause was determined to be flap displacement, partial dehiscence of the flap rim, or hematoma formation compromising the flap [28].

## 4. Indications, Modifications, and Postoperative Considerations

**Figure 3:**
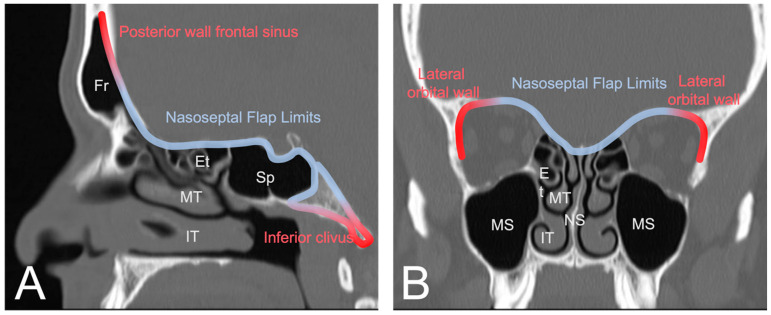
(**A**) Sagittal view of CT sinus without contrast through frontal sinus outflow tract demonstrating the general reach of the nasoseptal flap (blue). The posterior wall of the frontal sinus and the lower third of the clivus are typically too distal for the conventional nasoseptal flap (red). (**B**) Coronal view demonstrating lateral reach covering the ethmoidal roof and medial bony orbit (blue) with distal limits at the lateral orbital wall (red). Image from *Radiopaedia*.*org*. Fr: Frontal sinus; Et: Ethmoid air cells; Sp: Sphenoid sinus; MT: middle turbinate; IT: inferior turbinate; MS: maxillary sinus; NS: nasal septum.

The nasoseptal flap is the default reconstructive option for most anterior skull base defects. Several modifications to the original design have been proposed for when additional tissue or reach is needed. In this section, we will highlight some of these variations. Though the flap is very versatile with a robust arc of rotation and the ability to include a large mucosal surface, it has limited reach superiorly toward the posterior nasal wall of the frontal sinus, laterally toward the orbits, and inferiorly beyond the upper two thirds of the clivus [29] (Figure 3). Cadaver studies have shown that total sphenoidotomy reduces the mean length of the nasoseptal flap by increasing the distance to the posterior wall of the frontal sinus [30]. Therefore, several modifications have been proposed to extend its reach. 

To facilitate the coverage of anatomic areas such as the posterior table of the frontal sinus and inferior third of the clivus, several modifications have been proposed to extend the reach of the NSF. The vascular pedicle can be released laterally toward the sphenopalatine foramen with removal of overlying bone and extension of the inferior mucosal incision laterally across the choana above the eustachian tube to the medial pterygoid plate. Sacrifice of the vidian neurovascular bundle allows exposure for drill-out of the base of the pterygoid bone, posterior to the greater palatine canal [31]. Exposure of the pterygoid fossa through a maxillary antrostomy and subsequent drilling of the orbital, vertical, and sphenoid processes of the palatine bone may allow for the flap to extend further in the ventral direction. This technique has the added advantage of allowing additional nasal mucosa to be harvested to greatly expand the breadth of the flap [32,33]. 

**Figure 4:**
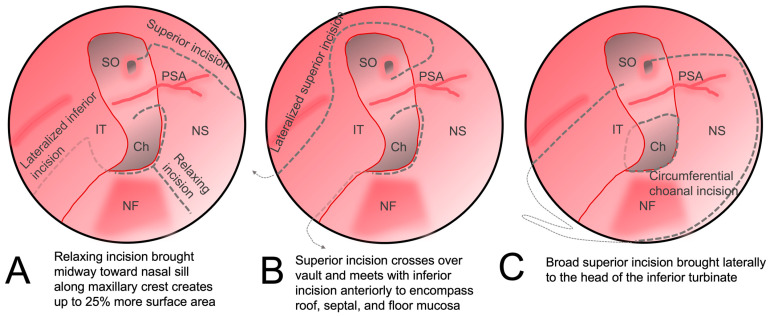
Alterations to the original nasoseptal flap design to increase coverage. (**A**) The inferior incision is lateralized to the nasal wall and third incision is made along the maxillary crest which augments the final surface area of the flap. (**B**) The superior incision is carried laterally superior to the sphenoid ostium and brought anteriorly along the inferior turbinate and then swung circumferentially across the nasal roof to meet with the inferior incision on the anterior septum. (**C**) The mucosa around the choana is released and then a superior incision is brought across the nasal floor to the head of the inferior turbinate to incorporate an inferior turbinate flap. IT: inferior turbinate; NF: nasal floor; NS: nasal septum; Ch: choana; PSA: posterior septal artery; SO: sphenoid os.

Additional reach can also be achieved by making additional mucosal cuts. A releasing incision along the maxillary crest from the posterior edge to the middle of the flap has been shown to increase the surface area of the flap (Figure 4A) [34]. To reach more laterally for the reconstruction of medial orbital wall defects, the anterior incision can be extended superiorly and outward laterally in a circumferential manner down the lateral nasal wall toward the inferior turbinate, allowing the mucosa of the entire ipsilateral septum and nasal floor to be included (Figure 4B) [35]. Alternatively, the NSF can be combined with the inferior turbinate flap (Figure 4C). The robust dual vascular supply from the posterior septal and lateral nasal arteries allows this large mucosal flap to be advanced over large defects [36].

Another option is to suture a free mucosal graft from the contralateral nasal cavity to the NSF [37]. 

In high-flow CSF leak repair, the NSF is often used as the vascularized portion of a multi-layer closure. In a series of 38 patients with large skull base defects, a fascia “button graft”, which is a single inlay/onlay graft created by suturing two pieces of autologous fascia lata together in the middle [38], was used for primary dural repair, and then covered with a NSF. This potentially avoided the need for a lumbar drain for CSF diversion, and no postoperative CSF leaks or cases of meningitis occurred [39]. The role of lumbar drains after reconstruction using NSFs remained undefined until recently, with some surgeons believing that the NSF avoids the need for a lumbar drain and allows for earlier ambulation and a reduced risk of meningitis [40]. However, in a recent randomized control trial in patients with high-flow anterior skull base defects, lumbar drains were shown to reduce the postoperative CSF leak rate from 21.2% in the control group to 8.2% in the lumbar drain group, the majority of which were closed using a NSF [2]. Importantly, there was no increased risk of meningitis. This was an important study providing level 1 evidence supporting the use of postoperative lumbar drains when high-flow leaks are encountered surgically, even when the NSF is used for the defect repair.

The NSF has also been shown to be useful in pediatric endoscopic skull base surgery. The theoretical concern in this population is that cranial growth may exceed facial growth; thus, the size of the NSF may be limited relative to the size of the skull base defect [41]. However, the NSF was successfully used in a series of 55 patients and reduced the CSF leak rate from 12.5% to 8.9% [42]. Another group reported 12 pediatric cases (age range 1-17 years old) with both benign and malignant anterior skull base pathologies, for which the NSF was used for reconstruction and only one postoperative CSF leak was reported. Importantly, there was no evidence of altered craniofacial growth during the follow-up period in these patients [43]. Partial middle turbinectomy may help with exposure of the NSF pedicle in younger patients with small cavities. The NSF was also used to successfully treat a frontonasal meningoencephalocele defect in the anterior skull base that had already failed four prior transcranial attempts at closure [44]. 

The indication for NSFs in endoscopic endonasal surgery has also expanded beyond their traditional use in covering skull base defects. They have been successfully deployed in the marsupialization of rathke cleft cysts, in which the floor of the cavity is lined with the NSF to create a drainage pathway for cyst contents [45]. Likewise, a similar method has been used for obliterating recurrent cholesterol granulomas in the petrous bone [46]. 

As discussed above, lumbar drains are an important consideration when high-flow CSF leaks are encountered, even when the NSF is used to close the defect. Other postoperative considerations include the use of antibiotics with central nervous system penetration to reduce the risk of meningitis and anti-staphylococcal coverage when non-absorbable nasal packing such as Merocel sponges (Medtronic Inc., Minneapolis, MN, USA) are placed. Stool softeners, antitussives, antiemetics, and guidelines for avoiding Valsalva maneuvers, nasal blowing, and strenuous activity should also be provided to patients [40,47]. To address nasal crusting, nasal saline is often started 1–3 days after surgery, and in-office debridement under endoscopic visualization is often scheduled to start two weeks after surgery and continued on a weekly or biweekly basis until the crusting has resolved. However, there are no comparative studies or consensus guidelines for this practice after NSF reconstruction, and most postoperative management is often institution- or surgeon-dependent.

## 5. Donor Site Complications

Donor site complications related to the NSF are also relatively common with a reported rate of 27% in a series of 121 patients who underwent primarily transsphenoidal surgery [28]. Crusting of the donor site is expected in the immediate postoperative period as the septal mucosa overlying bone and cartilage is denuded intentionally. Additional complications include septal perforation, cartilage necrosis, and prolonged crusting beyond 6 months. These issues typically arise beyond the immediate postoperative period and may be related to injury to the contralateral vascular supply to the septum during the procedure or due to prior septoplasty. However, no single risk factor has been identified. Rare complications included squamous metaplasia and nasal nose deformity. In comparison to patients who underwent endoscopic skull base surgery without the NSF, patients requiring the NSF report immediate ear pain and fullness within the first week after surgery, followed by nasal congestion and obstruction persisting for approximately 6 weeks. Fortunately, by 3 months, the sinonasal quality of life (as measured by Sinonasal Outcome Test-22, SNOT-22) returns to the baseline and no significant differences are seen between flap versus non-flap patients [48,49]. Younger patients may have a prolonged resolution of symptoms and should be encouraged to continue rigorous nasal hygiene through the postoperative period [50]. 

Interestingly, the use of a NSF did not affect the duration of nasal crusting, which may instead be related to the complexity of the surgical approach more than anything else [51]. However, in a small, nonblinded prospective study, the use of a free mucosal graft from the middle turbinate expedited mucosalization at the septal donor site (70% compared to 5% in controls at 3 weeks). At 6 weeks in the same prospective cohort, crusting was 5% in the graft group and 85% for the nongraft controls [52]. Likewise, in a series of 145 patients, use of the NSF was associated with earlier resolution of nasal crusting, whereas smoking and allergic rhinitis were associated with delayed resolution [53]. In summary, the comparative impact of the NSF on sinonasal symptoms such as crusting after surgery remains unclear, but may be related to differences in the size of the flap harvested and the closure technique used, with some groups emphasizing the need for mucosal grafts to facilitate re-mucosalization of the donor site. 

**Figure 5:**
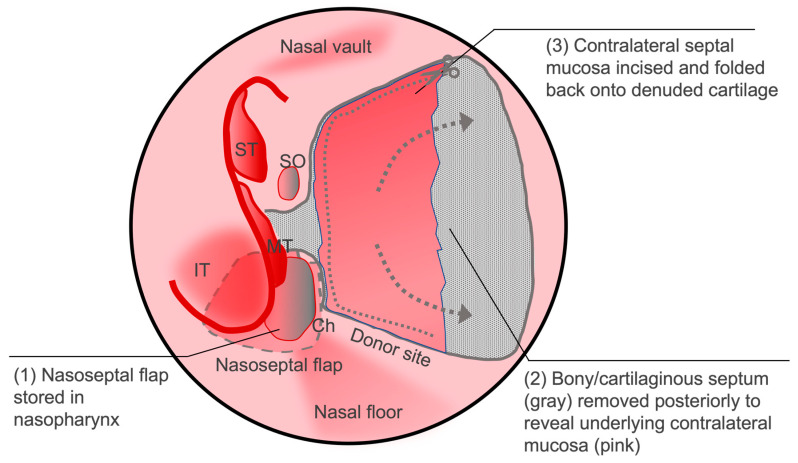
Depiction of the reverse flap to limit exposed septal cartilage and minimize crusting. After raising the conventional nasoseptal flap, a posterior bony septectomy is made, and the contralateral septal mucosa is incised and draped over the ipsilateral cartilage. IT: inferior turbinate; MT: middle turbinate; ST: superior turbinate; Ch: choana; SO: sphenoid os.

Another option to reduce postoperative donor site morbidity is the reverse rotation flap [54]. This technique involves performing a posterior septectomy by removing the ethmoid plate and vomer bone while preserving the contralateral septal mucosa. The contralateral mucosa is then incised to create an anteriorly based mucosal flap that is then draped over the ipsilateral donor site (Figure 5). In a review of 49 patients who received this reconstructive technique, 46 out of 47 patients had complete re-epithelization of the denuded area at follow up within 1–2 weeks with very few adverse events with just one patient each with granuloma formation, anterior dehiscence, and excoriated mucosa [55]. The reverse flap was also shown to decrease the incidence of external nose deformity following endoscopic sinus surgery in comparison to using the NSF alone [56].

Other complications that may occur after skull base surgery include complaints of nasal discharge, nasal obstruction, and anosmia/hyposmia. More rarely, patients may return with mucoceles or epistaxis. However, the contribution of the NSF itself as a risk factor for these sinonasal morbidities after endoscopic endonasal surgery is not known [51]. When nasal patency is measured quantitatively using acoustic rhinometry, the use of the NSF in transsphenoidal surgery did not impact postoperative values, and there was no strong association between the degree of nasal patency and quality of life outcomes to suggest that “empty nose syndrome” causes postoperative obstructive symptoms [57]. 

The impact of the NSF on anosmia differs greatly between studies, likely due to differences in technique. However, in a large study of 928 patients, the use of the NSF was the most significant risk factor for worse olfaction [58]. For this reason, a modified NSF was designed that leaves a generous margin of superior septal mucosa beyond the typical 1–2 cm olfactory strip, with the width of the flap instead taken from nasal floor mucosa. In a series of 40 NSF patients undergoing pituitary surgery in comparison to 58 control tumor cases (in which the olfactory region was not intentionally manipulated), no statistical differences in the degree of anosmia were noted, suggesting that when a large buffer of septal mucosa is left uninterrupted, postoperative smell loss from direct injury to the olfactory apparatus can largely be avoided [59]. 

External defects such as saddle nose deformity are relatively rare but have been reported after endoscopic endonasal surgery. In a review of 328 patients, 5.8% were noted to have postoperative dorsum collapse. The use of the NSF in reconstruction was the predominant risk factor; however, the extent of the resection and complexity of the reconstruction requiring larger NSFs likely play a role [60]. Interestingly, the authors of this study argue that the mechanism of collapse is likely not to be due to ischemic injury to the septum (which is more likely to result in septal perforation), but rather to stress on the cartilaginous septum and its attachments to the external framework during four-handed surgery.

Typical reconstructive ladders and algorithms dictate that intraoperative CSF leaks encountered during endoscopic transsphenoidal pituitary surgery warrant the use of the NSF [61]. The NSF is often combined with additional closure techniques such as continuous dural suturing and multilayered closure with autologous fascia grafts [62]. However, due to the sinonasal morbidity associated with the NSF, efforts have been made to de-escalate the complexity of the repair. In a series of 409 patients in which only a single-layer closure with a simple synthetic collagen dural inlay was used, including 135 patients with intraoperative CSF leaks, the postoperative CSF leak rate was 1.2%, comparable to that of the NSF [63]. If a CSF leak occurs around the inlay, or a high-flow leak is noted, a NSF is indicated [64]. When no intraoperative CSF leak is noted and the patient lacks a patulous sellar diaphragm, an oxidized cellulose-only technique is also sufficient [65]. These data suggest that in standard endoscopic pituitary surgery and in the absence of an unexpected high-flow leak, a single-layer closure without the use of the NSF is sufficient and avoids the morbidity associated with NSF harvest. An effective technique that reduces nasoseptal morbidity by avoiding the NSF, while still providing mucosal coverage of the defect, involves combining a collagen dural inlay with a free mucosal graft onlay harvested from the nasal floor/inferior turbinate [66]. 

## 6. Conclusions and Future Directions

The NSF has been the go-to endoscopic reconstructive option for anterior skull base defects since it was first developed almost 30 years ago. As endoscopic skull base surgery has progressed in the intervening years, many adaptations of the flap have been developed, and its limitations have been better defined. In this review, we touched upon alterations that can be made to the NSF to cover larger and more distal skull base defects. We discussed the impact that the NSF has had on postoperative CSF leaks as well as complications associated with the NSF and efforts underway to mitigate its sinonasal burden. 

In the future, virtual pre-surgical planning will help select who is a candidate for a NSF, and its ideal size and shape. For example, in a large analysis of craniofacial measurements, it was found that East Asians and females had a shorter nasoseptal:anterior skull base ratio, indicating that the NSF may not be suitable for repairing large or long defects in these patients without first characterizing them further [67]. In such scenarios, detailed radioanatomical measurements can be made to show the precise margin width of the NSF used in transsphenoidal, transethmoidal, and transclival approaches [68]. This type of approach can be manifested through 3D printed models of the sinonasal cavities based on preoperative CT imaging [69]. This may allow the surgeon to prepare the minimal flap needed to cover the predicted defect, with mucosal preservation in mind. 

## Figures and Tables

**Table 1:** Classification of anterior skull base defects.

**Location** [3]	Cribriform plate		
	Ethmoidal roof		
	Sphenoid		
	Frontal sinus		
	Sella turcica		
	Clivus		
**Size** [2]	<1 cm		
	>1 cm		
**Etiology** [3,4,5,6,7,8,9]	Iatrogenic	Planned	
		Accidental	Cribriform plate/fovea ethmoidalis
			Frontal
			Sphenoid
	Neoplastic	Meningioma, olfactory fossa, planum sphenoidale, tuberculum sellae	
		Craniopharyngeoma	
		Chordoma	
		Esthesioneuroblastoma	
		Sinonasal carcinoma	
		Sarcoma, chondrosarcoma	
		Benign	Osteoma
			Inverted papilloma
	Infectious/ Inflammatory	Invasive fungal sinusitis	
		Inflammatory pseudotumor	
		Osteomyelitis	
		Mucocele	
	Trauma	Sphenoid	
		Frontal	
		Cribriform plate/fovea ethmoidalis	
	Spontaneous	Congenital	
		Idiopathic	Benign intracranial hypertension
			Connective tissue disorder
**CSF leak** [1,2]	Low	Less than 1 cm dural defect, CSF weeping	
	High	Greater than 1 cm dural defect, involvement of ventricle or cistern	

**Table 2:** Risk factors for postoperative CSF leak.

After endonasal endoscopic skull base surgery [23]
Longer length of stay
Staged procedure
Preoperative hydrocephalus
Closure with graft (instead of vascularized flap)
Prior endoscopic skull base surgery [26]
Prior radiotherapy [26]
After closure with nasoseptal flap [5]
Abnormal BMI (<18.5 or >25)
Age > 65
Intraoperative CSF leak
Complex closure with combined open craniofacial approach
Surgeon experience [1]
